# A Case Report of Cardiac Failure in a Patient on Teduglutide for High-Output Ileostomy Stoma

**DOI:** 10.7759/cureus.37518

**Published:** 2023-04-13

**Authors:** Ambica Nair, Ankita Prasad, Shrujal Parikh, Moiuz Chaudhri, Vinod Nookala, Pramil Cheriyath

**Affiliations:** 1 Internal Medicine, Ocean University Medical Center, Hackensack Meridian Health, Brick, USA; 2 Internal Medicine, Rutgers Robert Wood Johnson Medical School, New Brunswick, USA

**Keywords:** glp 2, fluid overload, parenteral nutrition, bowel resection, ileostomy, short bowel syndrome, high output stoma, cardiac failure, teduglutide

## Abstract

A high volume of ileostomy output in patients with extensive bowel resection can be hard to manage. This leads to extensive loss of fluids and electrolytes along with malabsorption. Medications have traditionally controlled it by delaying intestinal transit and decreasing intestinal and gastric secretion using opiates, loperamide, diphenoxylate, omeprazole, somatostatin, and octreotide. However, many patients depend on parenteral nutrition and fluid and electrolyte infusions, even with optimal drug therapy. Despite the best possible care, they may develop renal failure. Teduglutide is a glucagon-like peptide-2 (GLP-2) analog given as a daily subcutaneous injection, and it has been promising in managing short bowel syndrome. It has been effective in decreasing the dependence on parenteral nutrition. However, improving fluid and electrolyte balance can precipitate cardiac failure in some patients, especially those with borderline cardiac functions, hypertension, and thyroid disorders. This usually presents in the first few months of the initiation of teduglutide therapy and may require stopping the medication. We present the case report of an elderly female with a high-output stoma on parenteral nutrition on teduglutide. There was a significant decrease in stoma output, and parenteral nutritional support could be stopped. However, she presented with worsening dyspnea and was diagnosed with cardiac failure with an ejection fraction of 16%-20%. The baseline ejection fraction was 45%, done six months before this. Coronary angiography showed no stenosis in any vessels, and the decline in left ventricular ejection fraction and fluid overload was attributed to teduglutide therapy.

## Introduction

Teduglutide is a synthetic derivative of glucagon-like peptide-2 (GLP-2). It is an intestinotrophic hormone secreted by enteroendocrine L cells of the intestinal epithelium. It increases epithelial proliferation, inhibits apoptosis, enhances barrier function, and increases digestion, absorption, and blood flow [1]. This helps to improve the absorption of nutrients and is used to treat short bowel syndrome (SBS). High-output ileostomy diarrhea where conventional therapy has been ineffective and parenteral nutrition is required [2]. These are conditions in which the body cannot absorb enough food due to a loss of functioning bowel. Teduglutide is administered as a subcutaneous injection, typically once daily. Several clinical trials have demonstrated teduglutide's efficacy in improving nutrient absorption and reducing dependence on parenteral support [2]. It also appears to be a treatment option for palliative care practice if patients suffer from SBS [3]. In one study, patients treated with teduglutide reduced their reliance on parenteral support by an average of 33% [4]. HOS patients have an activated renin-angiotensin system primed for fluid retention to prevent dehydration [5]. So, this improvement can also cause fluid and electrolyte imbalances and fluid overload, precipitating cardiac failure in patients [6].

## Case presentation

**Figure 1:**
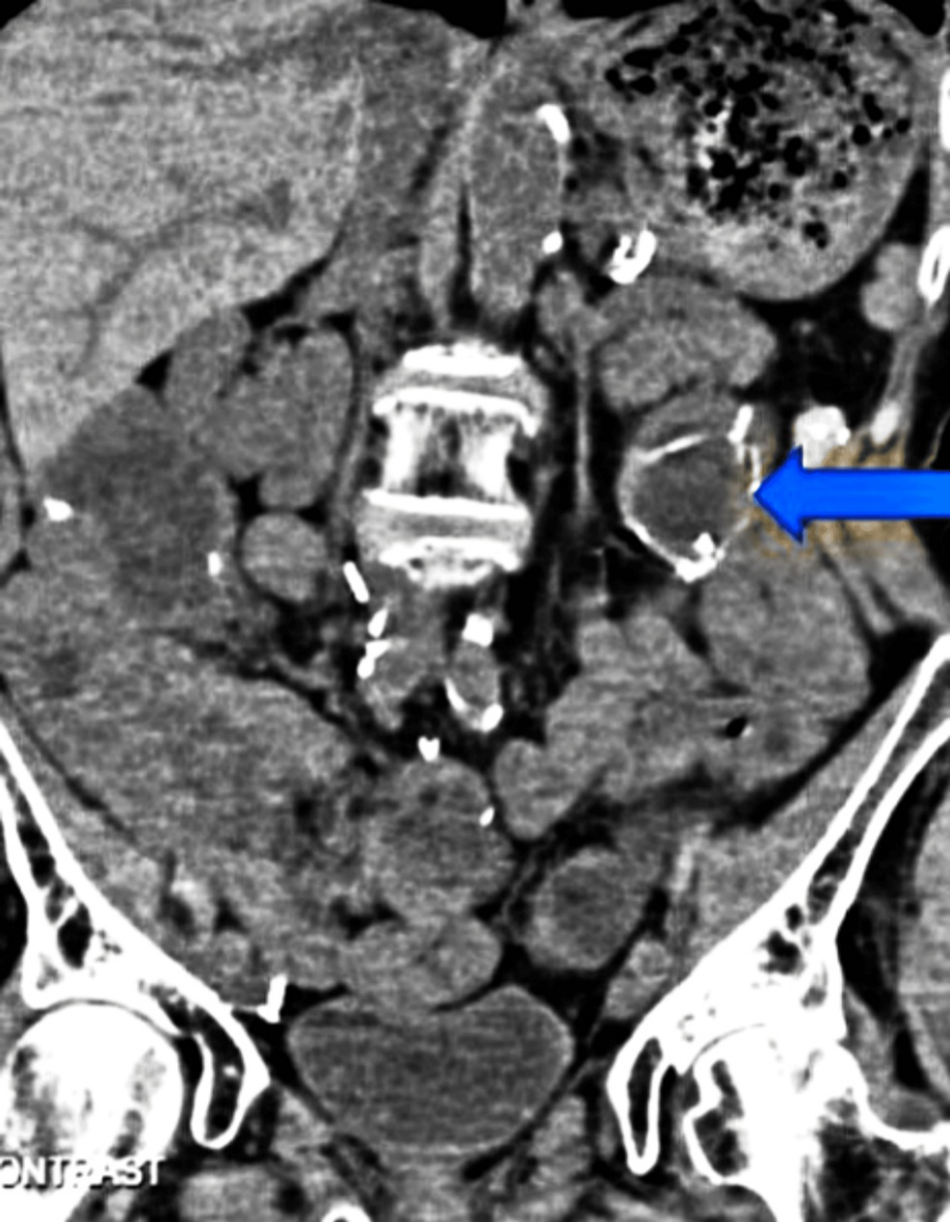
Contrast-enhanced computed tomography abdomen, coronal section with ileostomy (blue arrow)

Our patient is an 84-year-old female who presented with progressively worsening exertional dyspnea, productive cough, and swelling of both legs for one week. Her symptoms had worsened considerably over the last two days, and she was breathless with activities of daily living. She did not have similar complaints in the past. Her medical history was significant for chronic kidney disease (stage III) and anemia of chronic disease for which she was on iron and recombinant erythropoietin. Other pertinent past medical history included atrial fibrillation for which she was on apixaban, sick sinus syndrome status post pacemaker insertion, and endometrial carcinoma for which she had debulking surgery and associated adjuvant chemotherapy and radiation 20 years ago. She also had recurrent episodes of diverticulitis and colonic fistulas that required multiple abdominal surgeries over many years. She was diagnosed with moderately differentiated adenocarcinoma colon (T4N0), following which she had a colonic resection with a permanent L ileostomy bag (Figure 1).

The ileostomy was revised two years back due to a bleeding mass next to the ileostomy site. Since the revision of her ileostomy, she has had persistently elevated ileostomy output, due to which she had a protracted hospitalization of 40 days. She was initially treated with diphenoxylate/atropine and a tincture of opium. However, her stool output was persistently more than 20 liters per week. She was also started on weekly octreotide injections. She had several hospitalizations during this period due to prerenal acute on chronic renal failure secondary to volume loss from increased ileostomy output. She also received a short course in total parenteral nutrition and thrice weekly intravenous normal saline on an outpatient basis. In the interim, her chronic kidney disease progressed to stage 4 with a baseline creatinine of 1.8.to 1.9. She was thus started on daily injections of teduglutide, dramatically improving her ileostomy output. She continued receiving infusions and electrolyte repletion intermittently but had no episodes of prerenal acute on chronic renal failure. Two months before the presentation, the patient had stopped receiving infusions as her kidney function had stabilized at baseline without requiring additional hydration.

**Figure 2:**
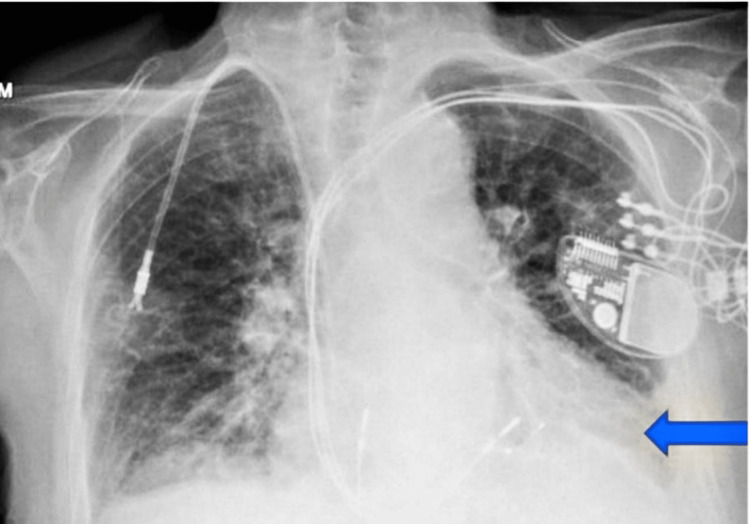
Chest x-ray showing pleural effusion (blue arrow)

The patient presented with shortness of breath after six months of daily teduglutide. For the last two months before the presentation, she was not receiving any fluid and electrolyte infusion. At the presentation, she was conscious and alert with respiratory distress. Her heart rate was 94/min, regular in rate and rhythm; her blood pressure was 124/83 mmHg, her respiration rate was 22/min, and her oxygen saturation was 94% on room air. Her ECG was normal, and the laboratory investigations showed a high BNP-664. Chest x-ray (CXR) is significant for mild bilateral pleural effusions (Figure 2).

Echocardiogram showed a moderately dilated left ventricle and left atrium. The left ventricular systolic function was severely reduced, with an ejection fraction of 16%-20% (Video 1).

**Video 1:** Echocardiogram post teduglutide showing ejection fraction of 16%-20%

The aortic valve was thickened and had moderate aortic valve regurgitation, moderate to severe mitral regurgitation, and moderate tricuspid regurgitation. An echocardiogram done six months back showed a mildly dilated left ventricle with an ejection fraction of 41%-45% (Video 2).

**Video 2:** Echocardiogram done pre tedugutide showing ejection fraction 40%-45%

The previous study also showed no evidence of regional wall abnormalities with mild aortic valve regurgitation with moderate mitral regurgitation. To investigate the cause of the reduction in left ventricular function, a nuclear stress test showed diffuse thinning with a severely enlarged left ventricle and blood flow, suggesting questionable apical and inferior apical ischemia. Inferior wall artifact limited evaluation; however, infarct at the inferior wall base with peri-infarct ischemia was suspected. There was also suspected ischemia involving the lateral wall. A single-photon emission computed tomography (SPECT)-gated wall motion study of the left ventricle was performed. The left ventricular ejection fraction was 28%, with wall motion showing hypokinesia. Coronary angiography was done to confirm the findings of ischemia. However, it showed no stenosis in the left main coronary artery, dominant large left anterior descending coronary artery, left circumflex coronary artery, posterior descending artery, and nondominant right coronary artery. The left ventricular end-diastolic pressure was measured to be 15 mmHg. Nonobstructive coronaries ruled out ischemia as a cause of the sharp reduction in left ventricular ejection fraction. The heart failure was attributed most likely due to teduglutide, which by reducing ileostomy output, resulted in severe volume overload.

After stopping teduglutide, she was started on furosemide, vericiguat, and carvedilol for her heart failure and her heart failure started improving symptomatically thereafter. She was also started on diphenoxylate-atropine and loperamide for managing her ileostomy output.

## Discussion

When established, the typical output of an ileostomy is 600-1200 mL per day [7]. When the outflow of the small intestine depletes water, salt, and often magnesium, a high-output stoma (HOS) or fistula develops. This usually occurs when the production exceeds 1.5 to 2.0 L/24 hours, although this varies according to the amount of food/drink ingested orally. Up to 31% of stomas in the small intestine demonstrate HOS [7]. If a small bowel causes HOS, the first step is to rehydrate the patient to relieve excessive thirst. When the normal renal function has been restored and thirst has diminished, oral hypotonic liquids are restricted, and a glucose-saline solution is sipped. Loperamide impedes intestinal transit in large doses, and omeprazole reduces gastric secretions so that it can lower stoma output. When less than 100 cm of functional jejunum remains, parenteral support is typically required [7]. Consideration should be given to restoring continuity to a defunct colon.

The fact that plasma levels of renin and aldosterone increase in ileostomy patients indicate that many are dehydrated and salt-depleted [8,9]. A urine sodium concentration of 10 mmol/L was found in 6%-13% of the population [10]. Chronic dehydration in these patients, if misdiagnosed, can lead to end-stage renal failure; hence, the creation of a chronic HOS must be detected early, treated well, and often monitored [7]. Diuretics use in HOS patients can hasten the development of renal failure and are best avoided.

Tinctures of opium and codeine phosphate slow down intestinal transit and have been used for many years to treat HOS. Diphenoxylate and loperamide have little to no impact on the central nervous system and are widely used to manage HOS. Loperamide is favored over codeine phosphate because it does not depress the central nervous system and is not addictive. Loperamide and codeine phosphate reduce intestinal motility, resulting in a 20%-30% reduction in water and sodium output from an ileostomy [11]. In reducing ileostomy fluid's weight and sodium content, oral loperamide is more effective than codeine phosphate, and the combined effect may be more significant [11].

Somatostatin and octreotide are other drugs used to manage HOS because they reduce saliva, gastric, and pancreaticobiliary secretions [7]. They also slow the movement of the small bowel and may delay the emptying of the stomach. Many patients have been able to decrease their need for parenteral nutrition; however, it is rare for patients to stop all parenteral fluids on these.

Teduglutide is a 33-membered polypeptide analog of GLP-2. Teduglutide and natural GLP-2 differ by one amino acid; glycine replaces alanine [12]. This keeps the dipeptidyl peptidase from breaking down the molecule, extending its half-life from seven minutes (GLP-2) to about two hours while keeping its pharmacological properties [12,13]. The pharmacological properties include maintaining the intestinal mucosa, increasing intestinal blood flow, decreasing gastrointestinal motility, and releasing stomach acid. They are believed to promote mucosal growth and restore stomach emptying and secretion [12,13]. It treats adults and pediatric patients one year and older with SBS requiring parenteral nutrition. It facilitates the intestinal absorption of nutrients and can assist in reducing the need for intravenous feedings or fluids. Studies have shown it to be safe, well tolerated, and intestinotrophic and suggested pro-absorptive effects facilitating reductions in parenteral support in patients with SBS with intestinal failure [12]. A metanalysis to find the efficacy of teduglutide by Fabio et al. found a response rate of 64% at six months, 77% at one year, and 82% at ≥ two years, and the weaning rate was 11% at six months, 17% at one year, and 21% at ≥ two years [4]. The response rate was defined as a 20% reduction in parenteral nutrition, and weaning meant independence from parenteral support [4].

Teduglutide's most common side effects (10%) are stomach pain, nausea, upper respiratory tract infection, abdominal distension, injection site reaction, vomiting, and hypersensitivity. Fluid overload has been observed in clinical trials and post-marketing. Congestive cardiac failure has been reported as a common adverse effect. If fluid overload occurs, especially in patients with cardiovascular disease, parenteral support, and teduglutide therapy should be reduced and reevaluated [14]. This is listed as a common complication of teduglutide with a frequency of 1/100 cases. Still, when looking for any similar case we have yet to find any similar case report. It occurred most frequently during the first four weeks of treatment and decreased over time [15]. Due to increased fluid absorption, patients with cardiovascular diseases, such as cardiac insufficiency and hypertension, should be monitored for fluid overload, especially during the initiation of therapy [16]. Patients should be advised to contact their physician in case of sudden weight gain, face swelling, swollen ankles, or dyspnea. If severe cardiac impairment occurs while taking it, the choice to continue treatment must be reassessed. In addition, quitting teduglutide may cause fluid and electrolyte imbalances. Fluid and electrolyte status should be monitored in patients who cease to use it. Due to the possibility of increased absorption, patients receiving concomitant oral medicines that require titration or have a narrow therapeutic index should be watched for adverse effects [16]. The concurrent medication may demand a reduction in dosage.

## Conclusions

Despite the best conservative management, a HOS can be hard to manage. The patients may need parenteral nutrition and frequent intravenous fluid and electrolyte infusion. These patients are also prone to chronic renal failure. Teduglutide is a synthetic analog of GLP-1 receptor agonist and can help these patients by improving fluid nutrition absorption. It can effectively reduce parenteral nutrition requirements and quality of life. However, this can also lead to increased fluid absorption and heart failure in patients with a history of cardiac disease. So, patients on teduglutide should be closely monitored for fluid overload symptoms. Patients should be asked to report early if they develop shortness of breath or clinical features of fluid retention.
